# Online genetic databases informing human genome epidemiology

**DOI:** 10.1186/1471-2288-7-31

**Published:** 2007-07-04

**Authors:** Angela J Frodsham, Julian PT Higgins

**Affiliations:** 1 Public Health Genetics Unit, Strangeways Research Laboratory, Cambridge, CB1 8RN, UK; 2 Medical Research Council Biostatistics Unit, Institute of Public Health, University of Cambridge, Cambridge, CB2 0SR, UK

## Abstract

**Background:**

With the advent of high throughput genotyping technology and the information available via projects such as the human genome sequencing and the HapMap project, more and more data relevant to the study of genetics and disease risk will be produced. Systematic reviews and meta-analyses of human genome epidemiology studies rely on the ability to identify relevant studies and to obtain suitable data from these studies. A first port of call for most such reviews is a search of MEDLINE. We examined whether this could be usefully supplemented by identifying databases on the World Wide Web that contain genetic epidemiological information.

**Methods:**

We conducted a systematic search for online databases containing genetic epidemiological information on gene prevalence or gene-disease association. In those containing information on genetic association studies, we examined what additional information could be obtained to supplement a MEDLINE literature search.

**Results:**

We identified 111 databases containing prevalence data, 67 databases specific to a single gene and only 13 that contained information on gene-disease associations. Most of the latter 13 databases were linked to MEDLINE, although five contained information that may not be available from other sources.

**Conclusion:**

There is no single resource of structured data from genetic association studies covering multiple diseases, and in relation to the number of studies being conducted there is very little information specific to gene-disease association studies currently available on the World Wide Web. Until comprehensive data repositories are created and utilized regularly, new data will remain largely inaccessible to many systematic review authors and meta-analysts.

## Background

Following the human genome project [1] and with the increasing efficiency and throughput of genotyping techniques, very high numbers of genetic variants can be examined for predisposition to disease [2]. Vast untapped resources of genotyping data sit in laboratories across the world, unlikely to ever be published due to natural tendency to better disseminate the more striking of these findings [3]. As the world of genetics moves into the era of whole genome association studies, the amount of data generated will increase still further [2].

Interpretation of the findings of genetic association studies is problematic, not only due to the selective reporting of findings, but also due to limitations of design, conduct, sample size, suboptimal analysis, and inconsistent findings across studies [4,5]. Systematic reviews and meta-analyses offer valuable means of assembling and synthesising the totality of evidence. They offer maximal power to detect true effects, the highest precision to estimate gene prevalences and gene-disease associations, and enable investigation of differences and inconsistencies across studies. However, when based solely on the available published literature they are dependent on what results have been reported, and publication-related biases may be substantial. To partly overcome this, data can be requested from primary investigators, although lack of response, changes in personnel, lack of access to archived data and unwillingness to share data can hamper such attempts. A preferable approach is for collaborative combined analyses by consortia of multiple studies [6]. The Human Genome Epidemiology Network (HuGENet) is promoting meta-analyses of genetic association studies [7], which all, to some extent, depend on information being available about which groups have examined which genetic variants.

One means of making genetic association information available is through online databases. A discussion paper published in 2000 recommended that, although resources for the provision of genomic information on the web were adequate, the availability of genetic *epidemiology *data was limited. This was in part blamed on the relative youth of the field of genetic epidemiology at the time [8].

Here we present findings from a systematic search for genetic epidemiology data available on the World Wide Web. Our primary motivation was to seek resources that would facilitate thorough systematic reviews or meta-analyses of gene prevalence or genetic association. We were interested both in identification of relevant studies and in availability of data that might not be published in journal articles. For genetic association information we further sought to evaluate the role of online databases as a supplement to information contained in MEDLINE, from the point of view of either a literature-based meta-analysis or in the preliminary stages of a collaborative combined analysis.

## Methods

We sought databases containing epidemiological information on gene prevalence or genetic association. Prevalence databases were determined as those with information on population prevalence of genetic variants without information on the evidence that such variants are involved in disease susceptibility or progression. Association databases were determined as those containing epidemiological information relating specific genetic variants to specific health or disease outcomes. To identify these we investigated the databases listed in the 2005 issue of the Nucleic Acids Research Database issue [9] and used those listed on the Center for Disease Control and Disease Prevention (CDC) Office of Genomics and Disease Prevention website [10]. We supplemented this with a search of the world wide web using the Google [11] search engine, using the search term "database (genetics OR genomics)(phenotype OR disease OR epidemiology OR association)" on the 14^th ^October 2005. Links from all databases identified were followed to identify further databases. We excluded general purpose reference databases (such as MEDLINE and EMBASE), databases primarily presenting information on genomics or proteomics without information on epidemiological studies, databases providing a resource for families and health care practitioners, and reported databases whose websites were found to be non-functional.

We produced a list of prevalence databases, and a list of databases addressing variants of a single gene. Databases including association information on more than one gene were the subject of detailed investigation. We extracted information from these on content, source of data, regularity of update, size of the database, accessibility, search functions, connections to other databases, administration and funding, using a pre-piloted pro forma. We developed a system of grading the database according to its potential utility within systematic reviews and meta-analyses, as a supplement to a standard search of MEDLINE. This 'Beyond-MEDLINE utility grade' runs from grade 1 for a database that includes only material available in MEDLINE (and therefore would be identified by searching MEDLINE alone) to grade 5 for a database making unpublished data available to the user.

The grade definitions are as follows:

### 1 Nothing novel

Database entries are equivalent to/links to MEDLINE records;

### 2 Novel information

Database entries are based on MEDLINE records, but with additional qualitative information, or otherwise available data (e.g. a specifically written summary, or results extracted from the cited paper);

### 3 Novel data

Database entries are based on MEDLINE records, but with additional quantitative information otherwise unavailable (e.g. updated results or unpublished association data);

### 4 Novel studies

Database enables identification of association studies not mentioned in MEDLINE records (e.g. non-MEDLINE-indexed report of an association study);

### 5 Novel studies and data

Database enables identification of association studies not mentioned in MEDLINE records AND includes association data from such studies (e.g. grouped data or individual patient data).

## Results

A total of 448 websites were investigated, excluding duplicates. Of these, 257 were excluded, 111 were classed as containing prevalence data, 67 were classed a specific to a single gene and the remaining 13 databases were classed as containing information from genetic association studies and contained information on more than one gene. These were examined in more detail. Lists of all databases, by category, are available on our website [12].

The prevalence databases contained information on the frequency of genetic variation in multiple genes, often in more than one population. If a database only contained information relevant to a single gene, then this was placed in the gene-specific subcategory. The majority of databases in the gene-specific subcategory contained only prevalence data but some contained information about gene- disease associations, though these were often limited to the rather older field of single gene disorders. Databases containing information on only a single gene were excluded from the utility grade analysis.

Thirteen databases contained information on genetic association studies in more than a single gene (Table 1 and Additional file 1). The majority of the extracted databases are freely available to the scientific community, although three (Asthma Gene Database, MedGene and PharmGKB) require users to register in order to use the website. Most databases had entries that were specifically linked to MEDLINE citations, and added little to the information available in the relevant MEDLINE record beyond a summary of key findings. Five databases contained summary results for unpublished data, indications that a particular gene had been analysed, or (in the case of PharmGKB), access to the genotype and phenotype data enabling further analysis. These five databases of greatest utility in systematic reviews and meta-analyses are, however, restricted to the disease areas of Alzheimers disease, cardiovascular disease, hereditary inflammation and fever, pharmacogenetics and type 1 diabetes.

**Table 1:** a table summarising the key information from the databases identified as containing information on genetic association studies. Further information is available in the Supplementary information section. No of entries refers to the approximate number of different study reports contained within the specified database.

**Name of Website**	Website URL	Brief Description	**Host/Funding**	**No. of Entries**	**Date of last update**	**Beyond-MEDLINE Utility Grade**	**Accessibility**	**Reference**
Alzgene		Regularly updated collection of published genetic association studies performed on Alzheimer Disease phenotypes, from database searches and journals' contents lists. Case and control data presented. Performs crude meta-analysis of odds ratios on request.	Various	>1000	Mar 2006	5	Freely available	[14, 20]
Asthma and Allergy Gene Database*		Database of published studies for phenotypes related to asthmatic disease. Databases of linkage and mutation information include results extracted from peer-reviewed publications	Institut für Epidemiologie, Munchen	>20,000	Dec 2003	3	Registered access only	[21, 22]
Cytokine Gene Polymorphism in Human Disease		Regularly updated database with Medline-based records from a systematic review of cytokine gene polymorphisms associated with human disease. Data extracted from two publications about the study	University of Bristol/Genes and Immunity	>100	Mar 2002	2	Freely available	[23-25]
GDPinfo†		Extensive information system including large published literature database (currently only Medline records), HuGE reviews, books and various types of reports.	CDC	>15,000	Mar 2006	4	Freely available	[13, 26]
GenAtlas		Regularly updated database of genes, phenotypes and references. Among numerous databases are brief sections on disorders associated with genes, with lists of citations. May be biased towards statistically significant results	Université René Descartes, Paris	>60,000	Feb 2006	2	Freely available	[27]
GeneCanvas		Database of cardiovascular candidate genes and their polymorphisms investigated at INSERM (Paris, France). Data include gene frequencies and linkage disequilibrium statistics	Inserm	>750	Oct 2005	4	Freely available	
Genetic Association Database		Database of human genetic association studies of complex diseases and disorders, based on Medline records. Data Extracted from publications.	NIH/National Institute on Aging and Center for Information Technology	>8000	-	2	Freely available	[28]
Human Obesity Gene Map Database		Database of obesity-related genes, including P values for association and references. Biased in favour of statistically significant results.	Pennington Biomedical Research Centre, Louisiana State University	>100	Mar 2005	2	Freely available	[29-34]
Infevers		Database of genetic associations in hereditary inflammatory disorders, with voluntarily submitted entries. Submissions are validated by an editorial board member,	Institut de Génétique Humaine, CNRS/European Union 5^th ^framework	>400	Aug 2005	4	Freely available	[35]
MedGene		Automated database of gene-disease association studies in Medline.	Havard Medical School	Unknown	Apr 2005	1	Registered access only	[36]
OMIM		Database of human genes and genetic disorders, containing textual information with links to MEDLINE and sequence records in the Entrez system, and links to additional related resources at NCBI and elsewhere.	Johns Hopkins University	>16000	Mar2006	2	Freely available	[37-39]
PharmGKB		Database of genomic data and clinical information from participants in pharmacogenetics research studies. Welcomes submission of primary data.	Stanford University/NIH	>700	-	5	Registered access only	[40]
T1DBase		Database of type 1 diabetes data, including information from collaborating laboratories. Some indication given of unpublished data	Institute for Systems Biology/JDRF International/JDRF/WT Diabetes and Inflammation laboratory	>200	Mar2006	4	Freely available	[41]

*- No longer being updated due to lack of financial support

† Reports included in the database may contain structured or unstructured data that are not from MEDLINE- indexed paper

## Discussion

Our study aimed to identify, via a systematic search, the readily identifiable databases that have been set up to disseminate genetic epidemiology information over and above that available via MEDLINE to the scientific community. While many databases have been set up to house information on prevalence of genetic variation, with some notable exceptions little progress has been made in the field of gene-disease association data. In the 13 databases we identified on gene-disease association, all but one provided at least some extra information unavailable via a MEDLINE search alone. However, the seven databases among these that gave access to previously unavailable *data *(i.e. a utility grade of ∁3) clearly include only a small minority of the genetic association studies that exist (for example, Lin et al [13] found over 15,000 articles) The most useful of the databases, i.e. those providing the most, previously unavailable, information were considered excellent examples of resources potentially useful in systematic reviews and meta-analyses, but were targeted to particular fields, such as Type 1 diabetes, Hereditary Fever, Alzheimer's disease or pharmacogenetics. The utility of one such database for meta-analyses is demonstrated by a recent paper on Alzheimer's [14].

Many of the genetic epidemiology databases cited in the 2000 paper [8] are no longer updated or no longer exist, due a lack of financial support. Efforts and funding are needed to facilitate the further development of online repositories that enable the dissemination of all findings into the public domain. Any new repositories will need to provide some assurance of suitable quality control. The Human Genome Epidemiology Network (HuGENet) maintains the Published Literature Database [13], which is currently based on MEDLINE records alone. We would be keen to see this developed into a more comprehensive resource in the way that the Cochrane Central Register of Controlled Trials attempts to includes all clinical trials [15]. Neither database is currently structured to link together reports from the same study.

In the wake of the Human Genome Project, with the advent of high throughput genotyping technology, the HapMap project, and now in the era of whole genome association studies, many thousands of genotypes and other data will be generated from epidemiological studies. Only a small minority of these will be reported in traditional journals, and the published literature will continue to provide a potentially biased resource of only the most exciting findings [16]. The Human Genome Epidemiology Network (HuGENet) is committed to encouraging the dissemination of negative findings into the public domain via collaborating with existing journals and setting up on-line journals that will make this process easier. The 'Journal of Negative Results in Biomedicine' published online by BioMed Central [17] has already published several sets of null results of genetic associations and other journals have dedicated subsections for the reporting of null results [18].

We would strongly encourage individual study investigators, and especially consortia of investigators such as those in the HuGENet network of networks [6], to assemble and maintain lists of studies and data repositories. To enable the latter, an approach similar to that of the microarray research community could be adopted for gene-disease association studies: the MIAME (Minimum Information About a Microarray Experiment) guidelines encourage provision of sufficient detail about a microarray experiment for it to be replicated, and offer a format for data to be held in public repositories. Until such developments, it will continue to be difficult to interpret findings from genetic epidemiological studies easily and to fully include them in rigorous and regularly updated meta-analyses.

Since the completion of this study, the National Center for Biotechnology Information (NCBI) have announced a new database called dbGaP specifically to host genotype-phenotype studies [19]. This database appears to be an ideal example of the sort of database for which we were searching and will hopefully, in time if adequately utilised, form an essential resource for those preparing systematic reviews and meta-analyses of gene-disease association studies.

## Conclusion

As a result of our systematic search for online repositories of genetic epidemiology data, we found 13 databases containing information on genetic association on more than one gene. On grading each of these with respect to the amount and type of extra data contained compared with a search of MEDLINE, we found seven that contained completely novel data that was previously unavailable (i.e. utility grade ≥ 3). This suggests that systematic reviews and meta-analyses based on published reports could be usefully supplemented with searches of some of these resources. However, the yield of information on the world wide web was still disappointingly low, and neither published literature nor online databases appear adequate to find all relevant evidence for inclusion in a comprehensive meta-analysis. We encourage study investigators to make their published and unpublished data available in suitable online repositories. A single resource providing structured data from genetic association studies covering multiple diseases would be an invaluable resource.

## Abbreviations

CDC Center for Disease Control

HuGENet Human Genome Epidemiology Network

## Competing interests

The author(s) declare that they have no competing interests.

## Authors' contributions

AJF participated in the design of the study, carried it out, and drafted the manuscript. JPTH conceived of the study, participated in its design and coordination and helped draft the manuscript. Both authors approved the final manuscript.

## Pre-publication history

The pre-publication history for this paper can be accessed here:



## Supplementary Material

Additional file 1 This file list all of the 13 extracted databases and gives a more detailed description of each Click here for file
