# Supplementary material for: Online genetic databases informing human genome epidemiology
Source: BMC Med Res Methodol. 2007 Jul 4;7:31. doi: 10.1186/1471-2288-7-31 (PMC1929117; doi:10.1186/1471-2288-7-31)
Supplement: Additional file 1 — This file list all of the 13 extracted databases and gives a more detailed description of each [file 1471-2288-7-31-S1.doc]

# Supplementary Information

**Alzgene**

This database is aimed at providing an unbiased, centralized, publicly available and regularly updated collection of genetic association studies performed on AD phenotypes. Data are extracted following systematic searches of publicly available scientific literature databases (NCBI's PubMed and ISI's Current Contents®), as well as the table of contents of journals in genetics, neurology and psychiatry. Extracted data include essential characteristics of the investigated populations (e.g. origin and ethnicity, sample sizes, onset ages, gender distribution) as well as gene-specific results and genotyping details. The only exception from sampling all available genetic association studies are the ε2/ε3/ε4 polymorphisms in APOE - the only established AD risk gene - for which only a subset of studies is included. Only studies published in peer reviewed journals are considered for inclusion into the database. The database can be searched either by gene/protein name or alias, as well as by chromosomal location. Summary overviews for each gene include population specific information, as well as study-specific information (e.g. genotype distribution and allele frequencies, links to the polymorphisms studied, etc. Finally, a meta-analysis based on crude odds ratio (OR) estimates using up-to-date analytic methods is calculated from the available case-control association data for each gene.

Alzgene is searchable by chromosome, gene, protein, polymorphism, study or keyword. A standard entry gives a table with a published study (with citation) per line of the table, summarising key information such as population, source, number of polymorphisms studied, some summary data for the AD cases and controls and the overall result. It is also possible to comment on an individual report. Meta-analysis using the data presented for each gene is also possible. Results for a polymorphism search returns summary tables of allele frequencies grouped by ethnicity.

# Asthma Gene Database

Shutdown at the end of 2003, the Asthma gene database contains data on genetic association in the asthma and allergy field. Only peer-reviewed data are included in the database. The data are stored in an object-orientated manner and can be retrieved by standard SQL language. It uses a text-based and a graphical front end for data selection and analysis. The menu itself is divided into various sections called ‘General’, ‘Database’, ‘Misc Info’ and ‘More’. The "General" sections contain some administrative matters: a short introduction to the database, a menu for registration and updating of registration information, addresses for collaborations and homepage of the administrator. The "Database" section contains the main linkage and mutation tables, a general database statistics overview, gene expression data, gene therapy trials, a mouse page, an overview on relevant patents and business news from company press releases. "Misc Info" leads to books, supplements, family, segregation, twin and adoption studies and articles concerning ethics of asthma genetic research. The section "More" links to asthma and genetics pages in the internet. You can scan also the whole website from the search page.

The database section is searchable by chromosome, polymorphism type, linkage map and strength of association. A typical result returns the chromosomal location, gene name, polymorphism name, phenotype, study citation and overall result.

**Cytokine Gene Polymorphisms in Human Disease**

This database was created to house the information from a systematic review of the association of cytokine polymorphisms with human disease and was updated twice after that.

As in each of the published articles, the data are presented in three tables. The first includes all discovered cytokine gene polymorphisms. In the left-hand menu these can be found on the ' List of Polymorphisms Page'. The second table states the effect of these polymorphisms in in-vitro studies. These can be found in the ' Effect on Expression' page. The third table details the in vivo disease association studies, which have been further divided into those about 'TNFa/LTa and' those for 'Other' cytokines have been combined. Therefore each heading on the left-hand menu will lead to a table containing research on both of these publications. The information from the first publication and that from Supplement 1 have been combined and, all the tables of Supplement 2 are found on their own dedicated page in the left-hand menu.

A typical table details a study per line containing information on the gene and polymorphism, the phenotype, the study citation and the overall result.

# GDP Info

The Genomics and Disease Prevention Information System (GDPInfo) is a database of all of the documents available on the Office of Genomics and Disease Prevention’s (OGDP) Web site as well as links to relevant documents on other sites. The purpose of GDPInfo is to provide access to information and resources for guiding public health research, policy, and practice on using genetic information to improve health and prevent disease. The target audience for GDPInfo is the public health professional but there are data and information that would be of interest to a range of people from researchers to the lay public. The information available is grouped into the following sections: Book and Book chapters; Conferences, courses or workshops; Fact sheets; gene reviews; genetic competencies; genetic test briefs and reviews; genotype prevalence tables; HuGE case studies, HuGE e-journals, HuGE fact sheets, HuGE published literature and HuGE reviews; online presentations; public health perspective series; relevant links and other reports.

**GenAtlas**

Founded in 1986, GENATLAS compiles the information relevant to the mapping efforts of the Human Genome Project. The articles are daily analyzed by annotators to update the GENATLAS database. Only the objects with a known cytogenetic location are retained.

GENATLAS contains three kinds of searchable databases, Genes, Phenotypes and References Each gene record is divided in 6 chapters: - General information, DNA information, RNA information, Expression and localization information, Proteins information and Pathology information

The ‘full text search’ of this database is searchable by DNA type, protein category, gene location, Gene symbol or name, OMIM id or full text query. A query result contains several possible options that maybe suitable. Clicking on the hyperlink of the required option takes you to that genes’ specific information page. This contains information on genomic features such as structure, expression and function. Additionally, there is a section called ‘associated disorders’, which contains information on phenotypes associated with this gene with a summary of the variant and phenotype. A link at the top of the page (citations) will take you to articles that support these findings.

Another possible way to access gene- disease association data from this site is to use the ‘all sections’ search. In the section ‘pathology’ gives you the option to search for gene- disease association literature.

A potential drawback of this database is that only positive findings seem to be reported and not exhaustively.

**GeneCanvas**

The Gene canvas website houses information under the following searchable indexes: -

Articles/supplements: supplements to published articles

Articles/short reports: results published online but not in journals

Articles/debates and Opinions: discuss hot topics

Links: external links to access interesting web sites

Downloads: to download software developed in this lab

Genes: all genes investigated with links to their polymorphisms and related experimental and statistical information

Studies: list of studies of interest to investigate the genetics of cardiovascular disorders

Polymorphisms: the complete list of polymorphisms

Miscellaneous: sequence data

A typical gene entry contains data on gene name and symbol, the old Locuslink ID (that will redirect to Entrez gene) the Gene bank accession number, the chromosome location, details of polymorphisms, studies, statistical data, some citations and a contact for more information. A typical statistical data section contains information on the polymorphisms (detailing the alleles), the allele frequencies, some pairwise LD estimates, haplotype frequency estimates and an estimation of heterozygosity.

**Genetic Association Database**

The Genetic Association Database is an archive of human genetic association studies of complex diseases and disorders. The goal of the database is to allow the user to rapidly identify medically relevant polymorphism from the large volume of polymorphism and mutational data, in the context of standardized nomenclature.

The data included are from published scientific papers. Study data are recorded in the context of official human gene nomenclature with additional molecular reference numbers and links. The information is presented as gene-centred. That is, each record is (a record) of a gene or marker. If a study investigated 6 genes for a particular disorder, there will be 6 records.

An advanced search of the database allows searching of one or more fields such as phenotype, disease class, chromosome, chromosome band, official gene symbol, gene name or gene comment, DNA position, SNP Ids, strength of association, references, allele author description or functional effects, polymorphism class, population, study size, submitter, Entrez gene ID, OMIM ID, Unigene cluster number, Ensembl, presence of association, reference details, or alternative gene ID.

Results are returned as a table with each study representing a line on the table. Details are returned for each of possible fields detailed above. Links are also provided to Gene Expert and Disease Expert which link to specialist databases and /or experts in the field. Further links are provided to BBID, PubMed, Entrez Gene, Gene Cards, SNP resources, genomic sequence viewing resources such as Ensembl, and Mapview, HapMap, Reference sequences, GDPinfo, and ASAP @ UCLA.

**Human Obesity Gene Map Database**

The obesity gene map database results from a project to review annually all markers, genes and mutations associated or linked with obesity phenotypes at Universite Laval in Quebec City, Canada. The first version covered the evidence published until the fall of 1994 and was published in the Proceedings of the 7th International Congress on Obesity held in Toronto. Subsequently, yearly editions of the Human Obesity Gene Map have been published in Obesity Research, the official journal of the North American Association for the Study of Obesity. The 11th version of the map covers the literature until the end of October 2004.

The Obesity Gene Map web site was developed to address the need to include increasingly detailed information on the location and properties of an increasing number of obesity-related genes. Data from the authors’ published reviews was used as the starting point for constructing the Obesity Gene Map database. This information was then extensively cross-referenced internally, then linked to external resources such as OMIM, LocusLink and GenBank.

The website contains several links to separate databases containing different information on human genetics and obesity. There are sections specific to information about single-gene mutations, Mendelian disorders, associations and linkages, in addition to other non-human sections. The association page contains a table of information, with each line representing the results for a particular gene, with information on gene name, location, the number of cases, the particular obesity phenotype, strength of association and phenotype. It is worth noting that only significant results are included in this database.

**INFEVERS**

INFEVERS or INternet periodic FEVERS website was developed as a specialist site to gather updated information on mutations responsible for hereditary inflammatory disorders: i.e. Familial Mediterranean Fever (FMF), TRAPS (TNF Receptor 1A Associated Syndrome), HIDS (HyperIgD Syndrome), MWS (Muckle-Wells Syndrome)/FCU (Familial Cold Urticaria)/CINCA (Chronic Infantile Neurological Cutaneous and Articular Syndrome). Contributors submit their novel mutations through a 3 step form. Depending on the disease concerned, a member of the editorial board is automatically solicited to overview and validate new submissions, via a special secured web interface. If accepted, the new mutation is available on the INFEVERS web site and the discoverer, who is informed by email, is credited by having his/her name and date of the discovery on the site. The INFEVERS gateway provides researchers and clinicians with a common access location for information on similar diseases, allowing a rapid overview of the corresponding genetic defects at a glance. Furthermore, it is interactive and extendable according to the latest genes discovered.

Seven different diseases, each with their own database, are accessible from the main page. Entry to the database itself results in a table of results with each representing one polymorphism in one study. Details are given for the polymorphism name, its location within the gene, the kind of polymorphism, the sequence variant, the technique(s) used to identify/ genotype the polymorphisms, RFLP details, protein variant details, the functional consequences of the variant, functional tests, the number of controls, disease related symptoms, associated phenotype, ancestry origin, reference (with links to abstract or Medline citation if published), and input date.

**MedGene**

The accelerated pace of biological research and the advent of genomics have resulted in the prodigious accumulation of data about genes and their relationship to disease. This information is scattered among millions of different records in sources such as Medline and OMIM. In order to rapidly and comprehensively summarize the biomedical literature, MedGene used an automated approach to assemble disease-gene co-citation matrices from the titles, abstracts and MESH terms of over 11 million Medline records and normalized these gene-disease relationships into rank order. The estimated false negative rate for this tool is 9.2%, whereas the false positive rate ranged from 22% to 30% depending on the strength of the association. The resulting database can generate lists of genes for use in high-throughput screening experiments, can create disease-specific micro-arrays, and can sort the results of gene profiling data.

Users can review:

1. A list of human genes associated with a particular human disease in ranking order

2. A list of human genes associated with multiple human diseases in ranking order

3. A list of human diseases associated with a particular human gene in ranking order

4. A list of human genes associated with a particular human gene in ranking order

5. The sorted gene list from other disease related high-throughput experiments, such as micro-array

6. The sorted gene list from other gene related high-throughput experiments, such as micro-array

On entry to the gene section, a menu is given for searching via gene name, symbol or locus ID. Genes are displayed ranked by a statistical term (of which there are several to choose). On displaying the results, a link is created to Pubmed, and entry to this then returns all of the citations relating to a particular gene and gene/disease in one search.

**OMIM**

This database is a catalogue of human genes and genetic disorders authored and edited by Dr. Victor A. McKusick and his colleagues at Johns Hopkins and elsewhere, and developed for the World Wide Web by NCBI, the National Center for Biotechnology Information. The database contains textual information and references. It also contains numerous links to MEDLINE and sequence records in the Entrez system, and links to additional related resources at NCBI and elsewhere.

Each OMIM entry has a full text summary of a genetic phenotype and/or gene and has links to other genetic resources such as DNA and protein sequence, PubMed references, mutation databases, approved gene nomenclature, and more. In addition, NCBI’s neighbouring feature allows users to identify related articles from PubMed selected on the basis of key words in the OMIM entry. Through its many features, OMIM is increasingly becoming a major gateway for clinicians, students, and basic researchers to the ever-growing literature and resources of human genetics.

OMIM can be searched via filed tags such as title, OMIM number, allelic variants, text, references, clinical synopsis, gene map disorder, contributors, chromosome, creation or modification dates. Options to limit within these last2, together with other limiting options result in a more precise search.

Results return a page of text dedicated to a particular gene, with sections on gene cloning, structure function and mapping, molecular genetics, population genetics, animal models, allelic variants and a list of references.

**PharmGKB**

The PharmGKB database is a central repository for genetic, genomic, molecular and cellular phenotype data and clinical information about people who have participated in pharmacogenomics research studies. Its aim is to aid researchers in understanding how genetic variation among individuals contributes to differences in reactions to drugs. The data include, but are not limited to, clinical and basic pharmacokinetic and pharmacogenomic research in the cardiovascular, pulmonary, cancer, pathways, metabolic and transporter domains. The contributors tab contains the links to all of the projects submitting data to the PharmGKB.

The PharmGKB welcomes submissions of primary data from all research into genes and genetic variation and their effects on drug and disease phenotypes.

Pharm GKB is searchable via 6 different fields, genes (all/with PharmGKB primary data/with genotype data/ with literature annotations), diseases (all/ diseases with primary PharmGKB data) pathways, drugs (all/ drugs with PharmGKB primary data), submissions (by project/ by submitter/ other) or variants.

Results for the gene searches return a page with information on the gene, the number of positions analysed, phenotype datasets, pathways, literature annotations and other information. The page also contains links to OMIM, Entrez Gene, UCSC and Ensembl genome browsers, GDB, GO, Gene Atlas, Gene cards, MutDB, Promolign, NCBI Reference sequences, SOURCE and Swissprot in addition to a link enabling the download of primary data.

**T1Dbase**

T1DBase is a public website and database that supports the type 1 diabetes (T1D) research community. It is being created by a joint effort between the Institute for Systems Biology, Juvenile Diabetes Research Foundation/Wellcome Trust Diabetes and Inflammation Laboratory and the Juvenile Diabetes Research Foundation International. T1DBase collects information from public sources and from collaborating laboratories, integrates this information, and presents it in a form that is useful for T1D researchers. The current data includes annotated genomic sequences for suspected T1D susceptibility regions; microarray data; functional annotation of genes active in beta cells; and "global" datasets, generally from the literature, that are useful for systems biology studies. The site also includes software tools for analyzing the data.

The search engine is one of the main entry points to T1DBase. The home page has a search box in the centre of the page, and each individual page on T1DBase has a search box in the red title bar on the right.

The search database currently contains:

- Genes from the Beta Cell Gene Expression Bank
- Genes from the rest of the T1DBase Website
- Type 1 diabetes candidate regions
- Markers from dbSNP

Genes can be searched via

- Beta Cell Gene Expression Bank identifiers: Unigene ID, Sequence ID, Gene Symbol, Affymetrix Probeset ID
- Gene identifiers: Gene Symbol, Unigene ID, Entrez Gene ID, HomoloGene ID, OMIM ID, EPConDB DoTS ID, RefSeq ID or Gene Cards ID
- Candidate Region identifiers: Candidate Region Name, Candidate Region band location, RGD QTL ID
- Marker identifiers: RS ID, SS ID, Population Name
- Currently, only single-identifier searches are supported (lists will be supported in the future)
- Search results will be returned in a table with a section for each object type, a link to the page about the object and a summary of available information.
